# Evaluation of a specialist service model for treating body dysmorphic disorder (BDD): application of National Institute for Health and Clinical Excellence Guidelines for BDD (NICE, 2006)

**DOI:** 10.1192/bjo.2021.848

**Published:** 2021-06-18

**Authors:** Anusha Govender

**Affiliations:** South West London & St George's Mental Health NHS Trust

## Abstract

**Aims:**

Body dysmorphic disorder (BDD) is still poorly recognised with a dearth of research into treatment. The OCD/BDD Service (South West London St. George's Mental Health NHS Trust) is a specialist service offering treatment for BDD using the National Institute for Health and Clinical Excellence (NICE) stepped care model for BDD as a basis for service provision.

This is the only known study to date to evaluate the implementation of the NICE guidelines recommended treatment for BDD in clinical practice. Furthermore, a sample of patients and clinicians were interviewed to elicit their evaluation of treatment.

**Method:**

A total of 48 patients with a primary diagnosis of BDD who were offered treatment between 2006 and 2018 were identified. Questionnaires routinely completed at time of assessment were Yale Brown Obsessive Compulsive Scale for BDD (YBOCS-BDD); Montgomery-Asberg Depression Rating Scale (MADRS); Beck Depression Inventory (BDI) and Sheehan Disability Scale (SDS). Assessments were conducted by clinicians with expertise in BDD. Clinical data including risks and sociodemographic information were collated and analysed. Data were examined with intention to treat analysis. Thematic Analysis (TA) was used to analyse data from semi-structured interviews conducted with ten clinical staff and seven patients regarding their experiences of treatment. Qualitative data were coded and themes identified.

**Result:**

Clinical data at assessment indicated impaired functioning plus high risks and substance misuse. There was a higher percentage of females (58%); average duration of BDD was 19.23 years. Clinical outcomes indicated significant improvements in the total sample from baseline on measures of BDD, depression and functioning (p = 0.001). Patients described their progress in terms of living skills, social interactions and quality of life. The main themes identified included the significance of the therapeutic relationship expressed by both patients and clinicians; the lack of early intervention and knowledge of BDD in healthcare.

**Conclusion:**

Current recommendations for treating BDD were found to be beneficial overall. However, there are patients who are non-responders and including experiences of patients’ and clinicians’ perspectives provided valuable insights into other options for treatment which are lacking and could enhance current recommendations where CBT and medication have not enabled progress. The young onset age with long duration, highlights the need for developing awareness of BDD so that it is not a hidden disorder.

